# Common Treatment, Common Variant: Evolutionary Prediction of Functional Pharmacogenomic Variants

**DOI:** 10.3390/jpm11020131

**Published:** 2021-02-16

**Authors:** Laura B. Scheinfeldt, Andrew Brangan, Dara M. Kusic, Sudhir Kumar, Neda Gharani

**Affiliations:** 1 Coriell Institute for Medical Research, Camden, NJ 08003, USA; andrew.brangan@gmail.com (A.B.); dkusic@coriell.org (D.M.K.); neda.gharani@coriell.org (N.G.); 2 Institute for Genomics and Evolutionary Medicine, Temple University, Philadelphia, PA 19122, USA; s.kumar@temple.edu; 3 Department of Biology, Temple University, Philadelphia, PA 19122, USA; 4 Center for Excellence in Genome Medicine and Research, King Abdulaziz University, Jeddah 21577, Saudi Arabia; 5 Gharani Consulting, Surrey KT139PA, UK

**Keywords:** pharmacogenomic, machine learning, adaptation, human evolution

## Abstract

Pharmacogenomics holds the promise of personalized drug efficacy optimization and drug toxicity minimization. Much of the research conducted to date, however, suffers from an ascertainment bias towards European participants. Here, we leverage publicly available, whole genome sequencing data collected from global populations, evolutionary characteristics, and annotated protein features to construct a new in silico machine learning pharmacogenetic identification method called XGB-PGX. When applied to pharmacogenetic data, XGB-PGX outperformed all existing prediction methods and identified over 2000 new pharmacogenetic variants. While there are modest pharmacogenetic allele frequency distribution differences across global population samples, the most striking distinction is between the relatively rare putatively neutral pharmacogene variants and the relatively common established and newly predicted functional pharamacogenetic variants. Our findings therefore support a focus on individual patient pharmacogenetic testing rather than on clinical presumptions about patient race, ethnicity, or ancestral geographic residence. We further encourage more attention be given to the impact of common variation on drug response and propose a new ‘common treatment, common variant’ perspective for pharmacogenetic prediction that is distinct from the types of variation that underlie complex and Mendelian disease. XGB-PGX has identified many new pharmacovariants that are present across all global communities; however, communities that have been underrepresented in genomic research are likely to benefit the most from XGB-PGX’s in silico predictions.

## 1. Introduction

There is a well-established contribution of genetic variation to drug response that has resulted in the expectation of personalized optimization of drug efficacy and the minimization of drug toxicity [1,2,3,4,5,6,7]. Unfortunately, there is also a well-documented ascertainment bias in the populations that have been included in genetic and genomic research to date [8,9,10,11]. As a result of recent human evolutionary history, the out of Africa migration and resulting population bottleneck, Europeans carry only a subset of human variation [12,13,14,15,16]. Given the overrepresentation of peoples of European descent in pharmacogenomic (PGx) research, there are likely to be a non-trivial number of variants that impact drug response that have not yet been identified, functionally characterized, or incorporated into clinical guidelines. This bias, therefore, limits the generalizability of results from genomic and PGx studies to all human populations [9,11,17]. Efforts to mitigate this bias will help ensure that communities of European descent are not the sole beneficiaries of PGx research findings [8,11].

An illustrative example of the implications of PGx ascertainment bias is the case of warfarin dosing. A variant in the gene calumenin (the rs339097 G allele), rare in individuals with European ancestry, increases the required therapeutic dose of the commonly prescribed blood thinner warfarin by up to 15% [18]. This variant, as well as other key variants in established genes such as CYP2C9*5, *6, *8, and *11, have been left out of several common dosing algorithms and, as a result, these predictive models perform poorly for individuals that carry these variants [19,20,21].

Computational or in silico prediction methods for PGx variants have the potential to alleviate PGx ascertainment bias. Several methods have been developed to predict pathogenic variants, variants thought to negatively impact protein function [22,23,24,25]. Li et al. [26] extended this computational prediction effort to develop a method for functional missense PGx variants, but found that PGx variants looked less like disease variants (which are thought to have been subjected to purifying selection) and more like neutral variants. More recently, Zhou et al. [27] applied an ensemble computational approach to predict deleterious PGx variants and successfully applied it to the minority subset of PGx variants with existing experimental data. Consistent with Li et al. [26], Zhou et al. [27] found that relaxing the requirement of evolutionary signatures of purifying selection improved the computational prediction of PGx variants.

Previous work by us and others has demonstrated the impact that positive selection has had on global human contemporary variation involved in immune response and metabolism [11,28,29,30,31]. Given the overlap between these gene categories and the genes involved in drug response, we present here a novel approach to in silico PGx variant prediction that leverages signatures of adaptation. Our computational approach is designed to mitigate ascertainment biases in PGx research and identify important PGx diversity that is currently missing from existing PGx resources. 

## 2. Materials and Methods

### 2.1. Samples and Data

Whole-genome sequencing data from the Phase 3 of the 1000 Genomes Project [13] were used to identify global missense variation in previously annotated pharmacogenes in PharmGKB [32]; more detailed information about the 1000 Genomes Project Phase 3 population samples can be found in Table 1. Clinical Pharmacogenetics Implementation Consortium (CPIC) gene annotation information was downloaded from CPIC (https://cpicpgx.org/genes-drugs/) and was last annotated on 25 March 2020. Pharmacogene variant annotation information was downloaded from PharmGKB (https://www.pharmgkb.org/downloads/) on 28 October 2019. These data were compiled manually by PharmGKB scientific curators [32]. All of the available human UniProt feature annotations (ftp://ftp.uniprot.org/pub/databases/uniprot/current_release/knowledgebase/genome_annotation_tracks/UP000005640_9606_beds/) were downloaded on 6 December 2019 in bed format. Evolutionary probabilities were calculated as previously described for the subset of missense variant positions present in PharmGKB annotated pharmacogenes and in the UCSD 46 species vertebrate alignment [33,34], and candidate adaptive polymorphisms (CAPs) were identified as previously described [25,29]. Evolutionary rate, evolutionary time span, SIFT (Sorting Intolerant From Tolerant), and PolyPhen2 values were extracted from the e-GRASP Resource [35]. Version 1.5 CADD (Combined Annotation Dependent Depletion) values were downloaded from http://cadd.gs.washington.edu/download [36]. In total, 38,686 1000 Genomes Project Phase 3 whole-genome sequencing missense variants located in 1076 PharmGKB pharmacogenes with evolutionary probabilities were retained for downstream analyses (Supplementary Materials Table S1).

### 2.2. Enrichment Testing

We used a publicly available human dataset of adaptive signatures [28] and tested for enrichment of annotated PharmGKB pharmacogenes using a permutation approach. More specifically, for each neutrality test statistic (iHS, XP-CLR, and D) we conducted 1000 permutations assuming 29,521 total genes (the number of genes within 100 kb of one of the Illumina 1M duo SNPs included in [28]). We used the R sample function without replacement (replace = FALSE) to randomly sample the respective number of adaptive signatures for each statistic (9593 iHS loci, 8636 XP-CLR loci, and 17,734 D loci, respectively, across all population samples). We retained the number of permuted adaptive signatures that were annotated in PharmGKB as pharmacogenes. We then counted the number of permutations that were equal to or more extreme than the actual number of PharmGKB pharmacogenes that overlapped adaptive signatures identified by each statistic. We additionally used the pnorm function in R to calculate an empirical P-value to measure whether the extent of overlap between the number of actual pharmacogenes and adaptive signatures is expected by chance given the permutation distribution.

### 2.3. Machine Learning Modeling

For each missense variant position, UniProt feature annotations were coded as present or absent, CAPs were coded as present or absent, global minor allele frequency ranging from 0 to 1 was included, evolutionary probabilities for reference and non-reference alleles ranging from 0 to 1 were included, evolutionary rate ranging from 0 to 57,405 was included, and evolutionary time span ranging from 0 to 2774 was included. The pharmacogenetic outcome was generated from existing PharmGKB annotation, such that each missense variant was annotated as a pharmacovariant or not.

The Caret package in R [37], including the associated randomForest [38] and xgboost [39] packages, were used for all machine learning PGx modeling. We partitioned the data into 70% for training and 30% for testing using the createDataPartition Caret function. We used the DMwR package [40] smote method to balance the training data (using the Caret trainControl function with sampling = “smote”), and performed 5-fold cross validation and 10 repeats for the following models using the Caret train function: random forest (method = ‘rf’), Logit Boost (method = ‘LogitBoost’), and XG Boost (method = ‘xgbTree’), which each offering classification-based modeling. Given our relatively higher confidence in ‘true positives’, we weighted the model evaluation on sensitivity (metric = “Sens”).

## 3. Results

### 3.1. Annotated PGx Variation Is Negatively Impacted by Ascertainment Bias

**Figure 1:**
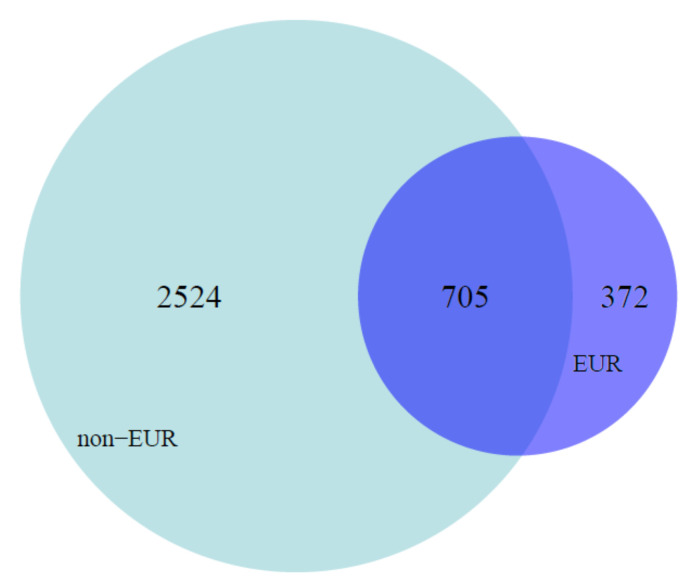
Venn diagram of 1000 Genomes Project Phase 3 pharmacogene variants.

To better characterize the potential impact of ascertainment bias on pharmacogene annotation, we performed a descriptive analysis of pharmacogenes annotated in CPIC (see methods for more detail) using the 1000 Genomes Project Phase 3 whole-genome sequencing data collected from worldwide populations (Table 1) [13]. We found that 70% of the genetic variants present in pharmacogenes annotated in CPIC are carried by non-Europeans, as displayed in Figure 1. This result is consistent with our expectation from global patterns of human genetic variation [12,13,14,15,16]. This result is also consistent with expectations from previous analyses of pharmacogene variation in worldwide populations [41] that the pharmacogene variation carried by Europeans alone is an incomplete picture of pharmacogene variation worldwide.

Figure 1 displays a Venn diagram of all of the single nucleotide polymorphisms (SNPs) included in the 1000 Genomes Project Phase 3 whole-genome sequencing dataset for all of the pharmacogenes that have at least one CPIC annotation. The light blue shaded area represents all of the variants present only in non-European population samples, the dark blue represents all of the variants present only in European population samples, and the overlapping area represents all of the variants present in both European and non-European population samples.

### 3.2. Pharmacogenes Are Enriched for Adaptive Signatures

Previous work by us and others has demonstrated the impact that positive selection has had on contemporary worldwide human variation involved in immune response and metabolism [11,28,29,30,31]. Moreover, in a study of 62 global human population samples, Li et al. [42] demonstrate signatures of positive selection in many pharmacogenes. To further explore the extent to which genome-wide signatures of adaptation are enriched for pharmacogenes, we leveraged the publicly available dataset of adaptive signatures identified in Scheinfeldt et al. [28]. This set of adaptive signatures was generated using three complementary approaches for the identification of adaptive signatures that are sensitive to classic selective sweeps and selection on standing variation and includes many genes known to play a role in immune response and metabolism across diverse African communities [28]. In this case, we have chosen to focus on signatures of past adaptation in Africa because our human ancestors emerged in Africa over two hundred thousand years ago and lived in Africa for tens of thousands of years before a subset migrated out of Africa over the past eighty thousand years; because of this bottleneck, non-Africans carry only a subset of human variation [12,13,14,15,16,28]. Consistent with Li et al.’s [42] results, our permutation enrichment test was significant for all three test statistics: iHS (*p* < 0.001), XP-CLR (*p* < 0.001), and D (*p* < 0.001). We found comparable results with our empirical P-value approach: iHS (empirical *p* < 0.001), XP-CLR (empirical *p* < 0.001), and D (empirical *p* < 0.001).

### 3.3. In Silico Model Development

Given the extensive pharmacogene variation in non-Europeans (Figure 1), the limited representation of non-Europeans in genomic and pharmacogenomic research to date, and the significant enrichment of pharmacogenes in adaptive signatures across the human genome, we next used a range of evolutionary statistics for each variable missense position in each pharmacogene (evolutionary rate, evolutionary time, evolutionary probability of the reference and non-reference allele, and whether the position contains a candidate adaptive polymorphism (CAP) according to Patel et al. [29]) together with global minor allele frequency and all available functional annotations included in the human subset of UniProt feature annotations to develop an in silico prediction method for functionally important pharmacogene variants (Table S1 includes more detail on the included pharmacogenes, and Table S2 includes more detail on the included pharmacogene variants).

We compared three machine learning model approaches and assessed which had the highest sensitivity to detect true positive pharmacogenes in a cross validation of both the training data and the testing data. Overall, the XG Boost model (XGB) performed the best on the training data (Table 2) as measured by ROC. While RF performed marginally better in terms of sensitivity (median 0.97 vs. 0.95, respectively), XGB performed significantly better in terms of specificity (median 0.70 vs. 0.45, respectively). The XGB model also performed better than the RF and LB models on the testing data with respect to sensitivity. As displayed in Table 3, XGB correctly identified more ‘true positive’ pharmacovariants annotated in PharmGKB (140 vs. 98 and 125, respectively, for RF and LB).

We additionally reviewed the variables that contributed to the XGB model. Table 4 includes the list of variables in order of importance. As shown, minor allele frequency (MAF) was the most impactful variable, followed by three evolutionary summary statistics: whether the position contains a CAP [25,29], evolutionary time [35], and the evolutionary probability of the non-reference allele [25]. The UniProtKB topological (Topo) domain feature (the location of non-membrane regions of membrane-spanning proteins) was the next most impactful variable, followed by evolutionary rate [35], the UniProtKB topological chain feature (the extent of a polypeptide chain in the mature protein), and the evolutionary probability of the reference allele [25]. Six additional UniProtKB features provide lower levels of impact on the XGB model.

### 3.4. Comparison with Existing Methods

Existing computational prediction methods have already been shown to perform poorly when applied to PGx data [43]. Our new XGB-PGX model outperforms SIFT, PolyPhen, and EVOD with respect to sensitivity, specificity, accuracy, and AUC (area under the receiver operating characteristic (ROC) curve) (Table 5). CADD performs marginally better with respect to specificity; however, XGB-PGX outperforms CADD with respect to sensitivity, accuracy, and AUC (Table 5). Given our lower confidence in our ability to identify ‘true negatives’, we consider the specificity results with additional caution.

### 3.5. Annotation Trends in PGx Variant Prediction

**Figure 2:**
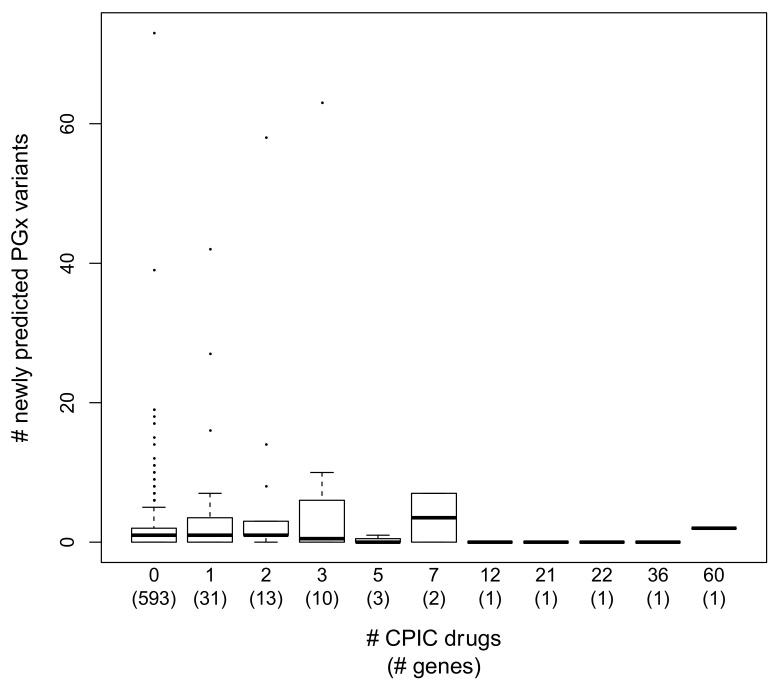
Boxplots of newly predicted pharmacogenetic variants across CPIC drug annotation categories.

We were interested in determining whether there were any trends involving the new XGB-PGX ‘predicted’ PGx variants. In particular, we asked if clinically well-studied pharmacogenes annotated in CPIC and PharmGKB have fewer ‘newly predicted’ PGx variants relative to pharmacogenes annotated in PharmGKB with less or no clinical annotation in CPIC. We reasoned that PGx variants in pharmacogenes that have been studied more extensively for clinical applications may be better understood than PGx variants in pharmacogenes that have been included in fewer clinical studies. We evaluated whether the PharmGKB pharmacogenes implicated in more CPIC drug-gene pairs have fewer ‘newly predicted’ PGx variants relative to pharmacogenes implicated in fewer CPIC drug–gene pairs, and used this comparison as a proxy to capture PGx variants in pharmacogenes that have been studied more or less extensively for clinical applications. Figure 2 displays the boxplot distributions of newly ‘predicted’ XGB-PGX pharmacogenetic variants for each category of drug–gene pair. While there is no exact linear relationship between the number of annotated CPIC drug/gene pairs and the number of newly ‘predicted’ PGx variants, pharmacogenes associated with more than 10 medications display a noticeable reduction in newly ‘predicted’ PGx variants: CYP2D6 (2 new), CYP2C9 (0), CYP2C19 (0), G6PD (0), ABCB1 (0). The full list of included genes, number of PharmGKB-annotated missense variants, number of newly predicted variants, number of putatively neutral missense variants, total number of variants included in the analysis, and total number of annotated CPIC drugs associated with each gene is included in Table S1. Table S2 includes variant-level information, including all of the variables included in the machine learning analyses, whether a given variant is annotated in PharmGKB, whether a given variant is a newly predicted pharmacogenetic variant according to XGB-PGX, and global minor allele frequency.

Figure 2 displays boxplot distributions of the number of newly predicted pharmacogenetic variants (along the *Y*-axis) for each category of pharmacogene (along the *X*-axis), each defined by the number of annotated CPIC drugs associated with a given gene. The *X*-axis labels denote the number of annotated CPIC drugs associated with a given gene category, and below in parentheses, the number of genes included in each category is included.

### 3.6. Allele Frequency Trends in PGx Variant Prediction

We were also interested in comparing allele frequency distributions between already known (PharmGKB annotated) and newly predicted pharmacogenetic variants, particularly given the impact that minor allele frequency had on the XGB-PGX model. If only a fraction of pharmacogenetic variation is known due to ascertainment bias, we would expect known pharmacogenetic variants to have relatively high allele frequencies in European population samples. To test this prediction, we calculated non-reference allele frequencies in each of the 1000 Genomes Project population samples.

**Figure 3:**
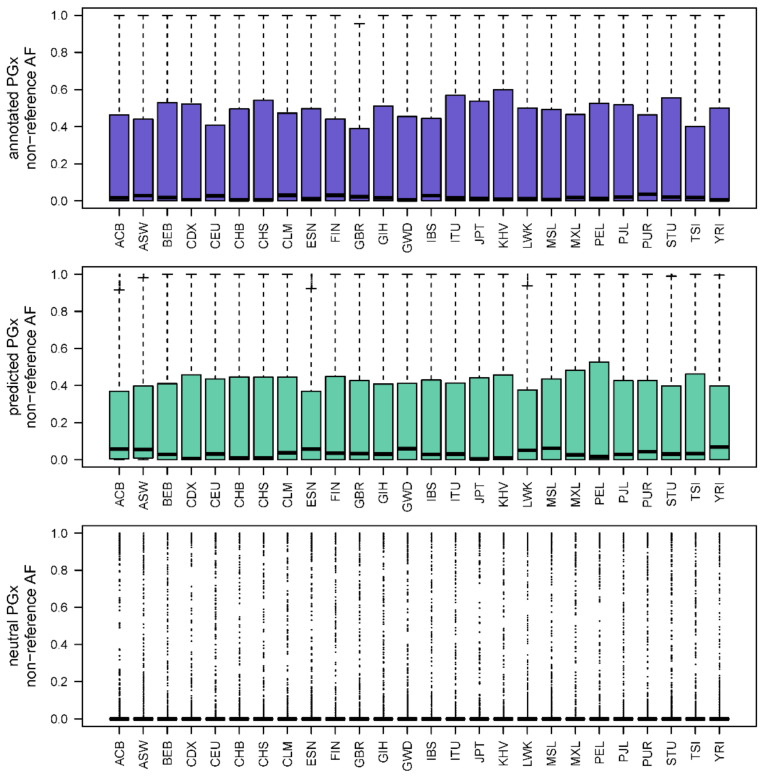
Allele frequency distributions across functional variant categories and population samples.

Figure 3 displays the distributions of PharmGKB annotated PGx variant allele frequencies, newly predicted PGx variant allele frequencies, and putatively neutral PGx variant allele frequencies across all 261,000 Genomes Project population samples. There do not appear to be meaningful differences in allele frequency distribution across population samples for already annotated pharmacovariants (Figure 3); however, XGB-PGX predicted variants are more common in African Caribbeans living in Barbados (ACB), people with African Ancestry living in Southwest USA (ASW), Esan living in Nigeria (ESN), Luhya living in Webuye, Kenya (LWK), Gambians living in Western Division, Mandinka (GWD), Mende living in Sierra Leone (MSL), and in Yoruba living in Ibadan, Nigeria (YRI). More notable is the dramatic increase in allele frequency in the annotated and predicted PGx variants relative to the putatively neutral variants.

The top panel of Figure 3 displays boxplot distributions of the non-reference allele frequency (along the *Y*-axis) of each PharmGKB annotated pharmacogenetic variant in each 1000 Genomes Project Phase 3 population sample (along the *X*-axis) in purple. The middle panel of Figure 3 displays boxplot distributions of the non-reference allele frequency (along the *Y*-axis) of each XGB-PGX predicted pharmacogenetic variant in each 1000 Genomes Project Phase 3 population sample (along the *X*-axis) in green. The bottom panel of Figure 3 displays boxplot distributions of the non-reference allele frequency (along the *Y*-axis) of each putatively neutral variant in each 1000 Genomes Project Phase 3 population sample (along the *X*-axis) in grey.

## 4. Discussion

The new in silico PGx variant prediction method, XGB-PGX, described here leverages identifiable adaptive signatures that have impacted missense variants across the human genome together with functional protein annotation information. Our approach is designed to mitigate ascertainment biases in PGx research and identify important global PGx diversity that is currently underrepresented or missing in existing PGx resources. This approach complements existing, annotated PGx resources and contributes to ongoing efforts to maximize drug efficacy and minimize drug toxicity in clinical care by identifying a more comprehensive set of PGx variants for functional characterization and clinical application.

XGB-PGX outperforms existing in silico functional variant prediction methods when applied specifically to PGx missense variation data. This performance improvement is likely due to the common assumption by existing methods that functional variants are deleterious and therefore rare in the general population. This assumption does not hold for PGx variation—presumably, at least in part, because of the documented impact of positive selection—and therefore needed to be adjusted in XGB-PGX for better performing PGx variant prediction.

We explored whether the number of newly predicted PGx variants followed any pattern related to clinical annotation. We found that CPIC annotated genes associated with seven or fewer medications had noticeably higher numbers of newly predicted PGx variants relative to CPIC annotation genes with more than ten associated medications. In particular, XGB-PGX identified no newly predicted PGx variants in ABCB1 (associated with 12 medications), CYP2C19 (associated with 21 medications), CYP2C9 (associated with 22 medications), and G6PD (associated with 36 medications), while XGB-PGX identified only two newly predicted PGx variants in CYP2D6 (associated with 60 medications). We interpret these results to suggest that the majority of the functional variation present in the most clinically studied pharmacogenes may already be known despite the ascertainment bias described above.

Interestingly, genes known to play important roles in immune response, such as the pharmacogenes that belong to the major histocompatibility complex (HLA-A, HLA-C, HLA-DQA1, and HLA-DRB1) have over 25 newly predicted missense PGx variants. Alternately, only one of the pharmacogenes (CYP4F2) belonging to the cytochrome p450 gene family (CYP2D6, CYP2B6, CYP2C9, CYP2C8, CYP2C19, CYP4F2), which is known to play a role in toxin metabolism, has more than two newly predicted missense PGx variants. These results suggest that further investigation of functionally predicted immune response variation is an intriguing new area for pharmacogenomic investigation.

We expected that our XGB-PGX prediction method would identify new PGx variants that would be more common in communities that have been underrepresented in PGx research. We found that the allele frequency distributions of already annotated and newly predicted PGx variants across 1000 Genomes Project global population samples include a range of allele frequencies, including both common and rare variation. We identified a modest increase in the newly predicted PGx variant allele frequencies in African Caribbeans living in Barbados (ACB); people with African Ancestry living in Southwest USA (ASW); Esan living in Nigeria (ESN); Luhya living in Webuye, Kenya (LWK); Gambians living in Western Division, Mandinka (GWD); Mende living in Sierra Leone (MSL); and in Yoruba living in Ibadan, Nigeria (YRI), as displayed in Figure 3. This trend is consistent with our initial assumption that existing PGx annotations are likely missing important variation, particularly in underrepresented communities (Figure 3).

The most striking difference among allele frequency distributions is between the relatively rare putatively neutral variants and the more common annotated and predicted functional PGx variants, regardless of population affiliation. The presence of a CAP at a given pharmacogene position is the second most important variable in XGB-PGX (Table 4), and this allele frequency pattern is consistent with our previous analyses of CAPs that demonstrated the majority of these adaptive variants to be common and shared across worldwide populations [29]. This pattern is also consistent with an older signature of adaptation that predates the out of Africa migration of modern humans [29]. More generally, these findings lend further support to a focus on individual pharmacogenetic testing rather than on presumptions about patient race, ethnicity, or ancestral migration history.

To date, a disproportionate amount of in silico modeling of functional variation implicated in disease and drug response has focused on rare, deleterious mutations [27,36,44,45]; however, we and others have demonstrated the important impact that positive selection has had in shaping variation at pharmacogenetic loci [28,29,42]. While negative or purifying selective pressure tends to suppress deleterious variation, positive or adaptive selective pressure tends to increase allele frequencies over time [46]. We therefore encourage more attention to be given to the important role that common genetic variation plays in pharmacogenomics and suggest a ‘common treatment, common variant’ perspective for pharmacogenetics that leverages the characteristics of pharmacovariants that are distinct relative to the deleterious genetic variants involved in disease.

While complementary to existing computational functional variant prediction methods that perform well in identifying rare, deleterious mutations involved in disease and drug response [27,36,44,45], there are several limitations to XGB-PGX. First, XGB-PGX is a predictive, in silico approach that requires functional validation and exploration of clinical relevance prior to any application to clinical interpretation. Second, XGB-PGX was developed using known pharmacogenes and the subset of missense variants that are in genomic regions that align to the vertebrate phylogeny; thus, variants located in alignment gaps will not be identified by our method. For example, none of the CYP2C9 and CYP2C19 variants that were functionally assessed by Devarajan et al. [47] were present in the aligned vertebrate phylogeny and the 1000 Genomes Project Phase 3 whole genome sequencing datasets used for XGB-PGX. In addition, XGB-PGX was trained on known PGx variants, and this subset is likely to be impacted by the same ascertainment bias we note above. We therefore have more confidence in true positives and less confidence in non-annotated ‘negatives’.

## 5. Conclusions

XGB-PGX has identified over 2000 new putative pharmacovariants that are equally relevant to worldwide communities regardless of geographic affiliation; however, communities that have been left out of past research may benefit the most from in silico prediction methods such as XGB-PGX until ascertainment bias in genomics and pharmacogenomics is solved.

## Figures and Tables

**Table 1:** 1000 Genomes Project Phase 3 data population samples.

Description	Label	Sample Size
African Caribbean in Barbados	ACB	96
Esan in Nigeria	ESN	99
Gambian in Western Division, Mandinka	GWD	113
Luhya in Webuye, Kenya	LWK	99
Mende in Sierra Leone	MSL	85
People with African Ancestry in Southwest USA	ASW	61
Yoruba in Ibadan, Nigeria	YRI	108
Colombians in Medellin, Colombia	CLM	94
People with Mexican Ancestry in Los Angeles, CA, USA	MXL	64
Peruvians in Lima, Peru	PEL	85
Puerto Ricans in Puerto Rico	PUR	104
Chinese Dai in Xishuangbanna, China	CDX	93
Han Chinese in Beijing, China	CHB	103
Japanese in Tokyo, Japan	JPT	104
Kinh in Ho Chi Minh City, Vietnam	KHV	99
Southern Han Chinese	CHS	105
British in England and Scotland	GBR	91
Finnish in Finland	FIN	99
Iberian Populations in Spain	IBS	107
Toscani in Italia	TSI	107
Utah residents (CEPH) with Northern and Western European ancestry	CEU	99
Bengali in Bangladesh	BEB	86
Gujarati Indians in Houston, TX, USA	GIH	103
Indian Telugu in the UK	ITU	102
Punjabi in Lahore, Pakistan	PJL	96
Sri Lankan Tamil in the UK	STU	102

**Table 2:** Machine learning model comparison using training data.

Statistic	Model	Minimum	1st Quartile	Median	Mean	3rd Quartile	Maximum
ROC	Random Forest	0.80	0.84	0.85	0.85	0.87	0.90
	LogitBoost	0.83	0.86	0.87	0.87	0.89	0.92
	XGBoost	0.88	0.90	0.91	0.91	0.92	0.94
Sensitivity	Random Forest	0.96	0.97	0.97	0.97	0.98	0.98
	LogitBoost	0.90	0.92	0.93	0.93	0.94	0.96
	XGBoost	0.93	0.94	0.95	0.95	0.95	0.96
Specificity	Random Forest	0.31	0.40	0.45	0.45	0.50	0.57
	LogitBoost	0.53	0.62	0.69	0.68	0.72	0.82
	XGBoost	0.61	0.67	0.70	0.69	0.72	0.78

**Table 3:** Machine learning model comparison using test data.

Model	Prediction	Not Annotated in PharmGKB	PharmGKB PGx
Random Forest	neutral	11,076	105
	PGx	326	98
LogitBoost	neutral	10,877	539
	PGx	525	125
XGBoost	neutral	10,716	63
	PGx	686	140

**Table 4:** Overall variable importance for XGB-PGx.

Variable	Overall Variable Importance (XGBoost)
Global minor allele frequency	100.00
Candidate adaptive polymorphism (CAP)	10.00
Evolutionary time	4.66
Non-reference evolutionary probability	1.81
Uniprot Topo domain	1.62
Evolutionary rate	1.21
Uniprot chain	1.16
Reference evolutionary probability	0.77
Uniprot domain	0.50
Uniprot helix	0.21
Uniprot repeat	0.18
Uniprot proteome	0.10
Uniprot disulfide	0.07
Uniprot variants	0.07

**Table 5:** PGx prediction performance comparison of in silico approaches.

Method	Sensitivity	Specificity	Accuracy	AUC
SIFT	0.59	0.42	0.50	0.51
PolyPhen2	0.60	0.44	0.52	0.53
CADD	0.73	0.78	0.75	0.56
EVOD	0.64	0.50	0.57	0.57
XGB-PGX	0.95	0.68	0.82	0.84

## Data Availability

Original/source data used in the analyses described in the paper are available as follows: 1000 Genomes Project Phase 3 whole-genome sequencing data are available at the following website: ftp://ftp.1000genomes.ebi.ac.uk/vol1/ftp/phase3/; Uniprot functional annotations can be accessed at the following website: ftp://ftp.uniprot.org/pub/databases/uniprot/current_release/knowledgebase/genome_annotation_tracks/UP000005640_9606_beds/; mypeg annotations can be accessed at the following website: http://www.mypeg.info/evod; PharmGKB annotations can be accessed at the following website: https://www.pharmgkb.org/downloads/; CPIC annotations can be accessed at the following website: https://cpicpgx.org/genes-drugs/; CADD values can be accessed at the following website: http://cadd.gs.washington.edu/download.

## Supplementary Materials

The following are available online at https://www.mdpi.com/2075-4426/11/2/131/s1: Table S1 includes the full list of genes included in the machine learning analyses, the number of PharmGKB-annotated missense variants, the number of newly predicted variants, the number of putatively neutral missense variants, the total number of variants included in the analysis, and the total number of annotated CPIC drugs associated with each gene. Table S2 includes all of the variables included in the machine learning analyses, whether a given variant is annotated in PharmGKB, whether a given variant is newly predicted pharmacogenetic variant according to XGB-PGX, and the global minor allele frequencies for all variants included in the machine learning analyses.
